# Causes of metabolic acidosis in canine hemorrhagic shock: role of unmeasured ions

**DOI:** 10.1186/cc6200

**Published:** 2007-12-14

**Authors:** Dirk Bruegger, Gregor I Kemming, Matthias Jacob, Franz G Meisner, Christoph J Wojtczyk, Kristian B Packert, Peter E Keipert, N Simon Faithfull, Oliver P Habler, Bernhard F Becker, Markus Rehm

**Affiliations:** 1 Clinic of Anesthesiology, Ludwig-Maximilians-University, Marchioninistrasse 15, 81377 Munich, Germany; 2 Department of Thoracic and Vascular Surgery, University of Ulm, Steinhövelstrasse 9, 89075 Ulm, Germany; 3 Department of General, Visceral and Thoracic Surgery, Clinic of Nuremberg, Prof.-Ernst-Nathan-Strasse 1, 90419 Nuremberg, Germany; 4 Sangart Inc., 6175 Lusk Blvd., San Diego, CA 92121, USA; 5 Alliance Pharmaceutical Corp., 4660 La Jolla Village Drive, San Diego, CA 92122, USA; 6 Clinic of Anesthesiology, Intensive Care Medicine and Pain Management, Krankenhaus Nordwest, Steinbacher Hohl 2-26, 60488 Frankfurt, Germany; 7 Department of Physiology, Ludwig-Maximilians-University, Pettenkoferstrasse 12, 80336 Munich, Germany

## Abstract

**Introduction:**

Metabolic acidosis during hemorrhagic shock is common and conventionally considered to be due to hyperlactatemia. There is increasing awareness, however, that other nonlactate, unmeasured anions contribute to this type of acidosis.

**Methods:**

Eleven anesthetized dogs were hemorrhaged to a mean arterial pressure of 45 mm Hg and were kept at this level until a metabolic oxygen debt of 120 mLO_2_/kg body weight had evolved. Blood pH, partial pressure of carbon dioxide, and concentrations of sodium, potassium, magnesium, calcium, chloride, lactate, albumin, and phosphate were measured at baseline, in shock, and during 3 hours post-therapy. Strong ion difference and the amount of weak plasma acid were calculated. To detect the presence of unmeasured anions, anion gap and strong ion gap were determined. Capillary electrophoresis was used to identify potential contributors to unmeasured anions.

**Results:**

During induction of shock, pH decreased significantly from 7.41 to 7.19. The transient increase in lactate concentration from 1.5 to 5.5 mEq/L during shock was not sufficient to explain the transient increases in anion gap (+11.0 mEq/L) and strong ion gap (+7.1 mEq/L), suggesting that substantial amounts of unmeasured anions must have been generated. Capillary electrophoresis revealed increases in serum concentration of acetate (2.2 mEq/L), citrate (2.2 mEq/L), α-ketoglutarate (35.3 μEq/L), fumarate (6.2 μEq/L), sulfate (0.1 mEq/L), and urate (55.9 μEq/L) after shock induction.

**Conclusion:**

Large amounts of unmeasured anions were generated after hemorrhage in this highly standardized model of hemorrhagic shock. Capillary electrophoresis suggested that the hitherto unmeasured anions citrate and acetate, but not sulfate, contributed significantly to the changes in strong ion gap associated with induction of shock.

## Introduction

During hemorrhagic shock, metabolic acidosis is common and conventionally considered to be due essentially to hyperlactatemia. The increase in blood lactate generally originates from both increased lactate production and reduced lactate metabolism. However, there is an increasing awareness that hyperlactatemia alone fails to explain the full extent of metabolic acidosis [1,2]. The presence of nonlactate, unmeasured anions has been suggested as an alternative marker of tissue hypoxia [3].

Traditionally, an elevated anion gap (AG) was thought to represent the presence of unmeasured anions. However, the AG can be confounded by lactate, electrolyte, and protein abnormalities [4,5]. Abnormalities of these plasma components are accounted for in the physicochemical approach to acid-base balance [6]. In this approach, the plasma weak acid concentrations (albumin and phosphate), the partial pressure of carbon dioxide (pCO_2_), and the strong ion difference (SID) (that is, the net charge difference between strong cations and strong anions) are identified as variables with independent effects on pH [6]. This technique will identify the presence of unmeasured cations or anions in plasma by calculating the strong ion gap (SIG) [7]. Moreover, the SIG has recently been identified as a powerful independent clinical predictor of mortality when it was the major source of metabolic acidosis [8].

The aims of this study, therefore, were twofold: (a) to determine the temporal profile of unmeasured anions in relation to other acid-base parameters on the basis of quantitative analysis in a highly standardized canine model of hemorrhagic shock and (b) to identify potential contributors to unmeasured anions. Capillary electrophoresis allows for both qualitative identification and then quantitative analysis of charged species in plasma. Candidates could be inorganic anions, such as sulfate derived from degradation of organic sulfates in tissue, and small organic anion intermediates of mitochondrial and cytosolic metabolism released into the extracellular space. Moreover, a healthy vascular endothelium is coated by an endothelial glycocalyx and this structure consists of large amounts of bound polyanionic heparan sulfates. During hemorrhagic shock, degradation of the endothelial glycocalyx might be associated with increased levels of circulating heparan sulfate and hence be an additional potential source of negatively charged species.

## Materials and methods

The results presented in this report originate from a comprehensive experimental study investigating the effects of a perfluorocarbon-based artificial oxygen carrier given to anesthetized dogs during resuscitation from hemorrhagic shock [9]. However, the aforementioned study does not contain data on acid-base balance, nor have these data been analyzed before. The investigation conforms to the *Guide for the Care and Use of Laboratory Animals *[10]. Licensure and approval of the investigation were obtained from the government of Upper Bavaria.

### Experimental protocol

The study was performed in 11 beagle dogs of either gender (weight 15.7 ± 1.1 kg). All animals were splenectomized at least 8 weeks prior to the experiment to exclude changes in red cell mass induced by splenic contraction during hemorrhage and acute anemia. Anesthetic management, surgical preparation, and insertions of different catheters have been described in detail elsewhere [9]. Briefly, after induction of anesthesia, mechanical ventilation was performed on room air to maintain normocapnia. Because of the large surgical wound area and because of a lack of heating in the ventilatory circuit, fluid losses required intravenous fluid replacement by an electrolyte solution containing 154 mmol/L Na^+ ^and 154 mmol/L Cl^- ^(15 mL/kg per hour), supplemented by 20 to 40 mmol potassium chloride. Core body temperature was kept at approximately 36°C with a warming pad and a warming lamp. After completion of surgical preparation, a 30-minute stabilization period was allowed to elapse before baseline control values were collected (time point: baseline, B). O_2 _consumption was measured noninvasively at 1-minute intervals using a Deltatrac metabolic monitor (Deltatrac II MBM-200; Datex-Ohmeda, part of GE Healthcare, Little Chalfont, Buckinghamshire, UK) connected to the respirator. Subsequently, all animals were hemorrhaged to a mean arterial pressure of 45 mm Hg. At all times during hemorrhage, the actual O_2 _consumption value was subtracted from the baseline value, and by use of a computer program, the actual integrated O_2 _debt was determined as a function of body weight [9]. Mean arterial pressure was kept at 45 mm Hg by stepwise withdrawing and reinfusing whole blood until a standard O_2 _debt of 120 mL/kg had been achieved. The induction of a standardized metabolic insult with an accumulated O_2 _debt of 120 mL/kg results in reproducible tissue hypoxia and a predictable mortality of 50%, which comes very close to clinical practice [11,12]. The blood was reserved for reinfusion and was stored with a CPDA (citrate, phosphate, dextrose, and adenine) additive (Compoflex; Biotrans GmbH, Dreieich, Germany) at 10% vol/vol.

After the standardized induction of shock, a second set of measurements was obtained (time point: shock, Sh) and the fractional inspiratory O_2 _concentration was increased to 1.0. Thereafter, for restoration of tissue perfusion, a 6% pentastarch solution (6% hydroxyethyl starch, 200/0.5; Fresenius SE, Bad Homburg, Germany) containing 154 mmol/L of sodium and 154 mmol/L of chloride was given at a dose equal to the volume of shed blood. A third measurement was performed after completion of resuscitation (time point: post-treatment, pT). Additional measurements were performed 30, 60, and 180 minutes post-therapy (time points: 30', 60', and 180', respectively). The animals did not receive any acetate-containing solutions.

### Blood sampling and analysis

Arterial blood samples were collected in blood gas syringes containing lithium heparin (Rapidlyte; Bayer Diagnostics, Fernwald, Germany) at B, Sh, pT, 30', 60', and 180'. These were immediately analyzed for pH, pCO_2 _(standard electrodes), and the plasma concentrations of sodium (Na^+^), potassium (K^+^), calcium (Ca^2+^), magnesium (Mg^2+^), chloride (Cl^-^) (ion-selective electrodes), and lactate (Lac^-^) (enzymatic method, quantification of H_2_O_2_), all integrated in a blood gas and electrolyte analyzer (Rapidlab 860; Bayer Diagnostics) and measured at 37°C. Additionally, serum phosphate (Phos) (UV photometry of a phosphomolybdate complex) and albumin concentration (Alb) (colorimetry of bromocresol complex) were measured using the same blood samples. Values for standard base excess and bicarbonate (Bic^-^) were derived by the blood gas analyzer.

Additional arterial blood samples were drawn into serum monovette tubes at the above-mentioned time points for capillary electrophoresis and determination of heparan sulfate concentrations. Serum was rapidly separated by centrifugation at 2,000 *g *for 10 minutes and was stored at -80°C until assayed.

For each sample, an apparent strong ion difference (SID_a_) was calculated:

SID_a _= (Na^+ ^+ K^+ ^+ Ca^2+ ^+ Mg^2+^) - (Cl^- ^+ Lac^-^).

The amount of weak plasma acid (A^-^) was calculated [13]:

A^- ^= [Alb] × (0.123 × pH - 0.631) + [Phos] × (0.309 × pH - 0.469).

The effective strong ion difference (SID_e_) was calculated [13]:

SID_e _= 1,000 × 2.46 × 10^-11 ^× (pCO_2_/10^-pH^) + A^-^.

To quantify unmeasured charges, an SIG was calculated [7]:

SIG = SID_a _- SID_e_.

The traditional AG was also calculated:

AG = (Na^+ ^+ K^+^) - (Cl^- ^+ Bic^-^).

The AG corrected for albumin and lactate (AG_corr_) was calculated [14]:

AG_corr _= AG + 2.5 × (4 - [Alb]) - Lac^-^.

### Capillary electrophoresis

A capillary electrophoresis system (Waters Chromatography, Division of Milipore, Milford, MA, USA) was used with UV detection of solutes at 214 nm. Separations were obtained on a fused-silica capillary (length, 60 cm; internal diameter, 75 μm) (Waters) or on a polyvinyl alcohol (PVA)-coated capillary (length, 60 cm; internal diameter, 50 μm) (Agilent Technologies, Böblingen, Germany). To prepare the samples for assay, 10 μL of serum was mixed with 990 μL of distilled water (dilution 1:100). In the case of the first type of capillary, an inorganic anion buffer for capillary electrophoresis (pH 7.7) (Agilent Technologies) was used. Samples were loaded hydrostatically for 30 seconds. Separations were conducted at a constant voltage of 20 kV. Under these conditions, a current of 15 μA was encountered while samples were running. All data were recorded on a computer with Millenium software (Waters Chromatography, Division of Milipore, Milford, MA, USA).

**Figure 1:**
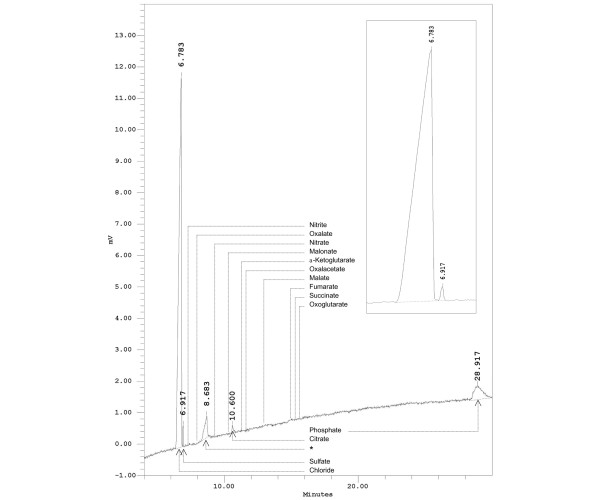
Analysis of anions in canine serum using a fused-silica capillary. As can be seen in the insert, the two anions, chloride and sulfate, migrated as distinct peaks, completely resolved from one another. The retention times for other inorganic and organic anions not detected in canine blood serum are indicated. The detection limits for sulfate and citrate were approximately 0.1 mmol/L. The conditions were as follows: fused-silica capillary (60 cm × 75 μm internal diameter); inorganic anion buffer, pH 7.7; running voltage, 20 kV; 25°C; detection: UV light transmission at 214 nm; sample: canine serum diluted with distilled water (1:100). Asterisk indicates unknown component introduced into serum from the 'coagulation' vial.

A typical electropherogram of a canine serum sample obtained with the fused-silica capillary is depicted in Figure 1. The high concentration of chloride in plasma resulted in a large positive peak. The identification of three further peaks was achieved by comparison with migration times of aqueous calibrators and spiking of the actual serum samples with stock solution of standard substances. Retention time and spiking identified the peaks as sulfate, citrate, and phosphate. A fourth prominent peak was an unidentified contaminant introduced into serum from the 'coagulation' monovette. Calibration curves based on quantification of peak areas were constructed using standard solutions of sulfate and citrate.

**Figure 2:**
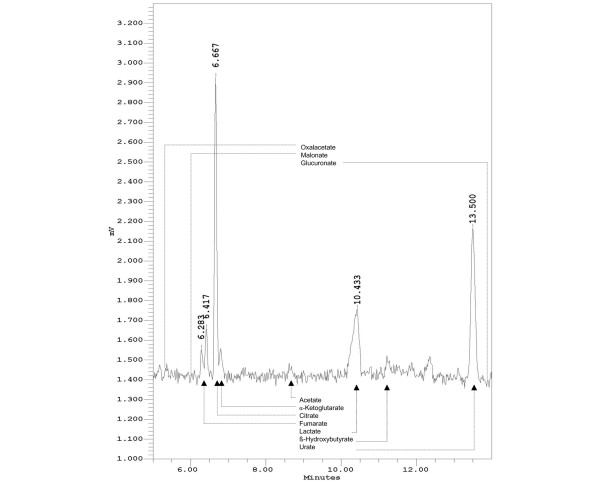
Analysis of anions in canine serum using a polyvinyl alcohol (PVA) capillary. The retention times for some other organic anions not detected in canine blood serum are indicated. The peak at 6.283 minutes remains unidentified. The detection limits were as follows: acetate 1.0 mmol/L, α-ketoglutarate 10.0 μmol/L, citrate 0.1 mmol/L, fumarate 1.0 μmol/L, β-hydroxybutyrate 0.7 mmol/L, and urate 0.1 μmol/L. The conditions were as follows: PVA capillary (60 cm × 50 μm internal diameter); phosphate buffer, pH 7.0; running voltage, 20 kV; 25°C; detection: UV light absorption at 214 nm; sample: canine serum diluted with distilled water (1:100).

A second type of capillary (PVA capillary), fitted with a 'bubble' in the optical window, provided higher sensitivity of detection, albeit other retention times and poorer separation of some anions of interest. A phosphate buffer for capillary electrophoresis (pH 7.0) (Agilent Technologies) was used. Figure 2 shows a typical electropherogram of a canine serum sample obtained with this type of capillary. The identification of seven peaks succeeded, again by comparison with migration times of aqueous calibrators and spiking of the serum samples. The peaks were identified as acetate, α-ketoglutarate, citrate, fumarate, lactate, β-hydroxybutyrate, and urate. Calibration curves based on quantification of peak areas were performed using aqueous calibrators of known concentrations.

### Measurement of heparan sulfate concentration and alkaline hydrolysis

Heparan sulfate concentrations were measured after pretreatment of serum with Actinase E (Sigma-Aldrich, St. Louis, USA) by using an enzyme-linked immunosorbent assay (Seikagaku Corporation, Tokyo, Japan). Additionally, serum samples were boiled with 1.0 M NaOH for 2 hours and serum sulfate concentrations were subsequently analyzed using capillary electrophoresis (see above).

### Statistical analysis

All data are presented as mean ± standard error of the mean. For normally distributed data (tested by Kolmogorov-Smirnov test), comparisons were made using analysis of variance for repeated measurements. For data that were not normally distributed, comparisons were made using analysis of variance on ranks. *Post hoc *testing was performed using the Student-Newman-Keuls method for multiple comparisons. Correlation between variables was evaluated using Pearson's product moment correlation. Differences were considered significant at a *p *value of less than 0.05.

## Results

One animal died from myocardial failure during shock induction and two animals dropped out during resuscitation and observation due to premature death, leaving eight dogs for final statistical analysis. Measured and calculated values of the acid-base status throughout the course of the experiment are presented in Table 1. During induction of shock, arterial pH decreased significantly from 7.41 to 7.19. An additional decrease in pH to 7.13 was observed after completion of resuscitation. pH had increased at 30, 60, and 180 minutes after therapy but remained lower than the baseline value. pCO_2 _increased transiently at the end of resuscitation and 30 minutes after therapy. It did not show major deviations from baseline at other times of the protocol. Directional changes in base excess were similar to changes in pH. Plasma concentrations of sodium, potassium, calcium, and magnesium did not show major deviations from the respective baseline values. The plasma concentration of chloride increased significantly 60 and 180 minutes after therapy. Serum lactate increased significantly from 1.5 mEq/L (baseline) to 5.5 mEq/L after shock induction and remained elevated until 30 minutes post-therapy. The SID_a _decreased significantly after completion of resuscitation and remained so post-therapy. The serum concentration of phosphate did not show major deviations from baseline. Due to hemorrhage and dilution with colloid solutions, the serum concentration of albumin decreased significantly after shock induction and remained decreased 30, 60, and 180 minutes post-therapy. The SID_e _decreased earlier than the SID_a _(after induction of shock) and remained significantly decreased until the end of the experiment.

**Table 1:** Measured and calculated values of the acid-base status

	Time point of measurement					
	Baseline	Shock	Immediately after therapy	30 minutes after therapy	60 minutes after therapy	180 minutes after therapy

pH	7.41 ± 0.01	7.19 ± 0.02^a^	7.13 ± 0.02^a^	7.23 ± 0.01^a^	7.27 ± 0.01^a^	7.26 ± 0.01^a^
pCO_2_, torr	33.4 ± 1.4	32.5 ± 2.6	43.8 ± 2.6^a^	39.4 ± 1.5^a^	36.4 ± 2.1	33.8 ± 1.0
sBE, mEq/L	-3.2 ± 0.5	-15.0 ± 1.0^a^	-14.7 ± 0.6^a^	-10.8 ± 0.7^a^	-9.5 ± 0.6^a^	-11.2 ± 0.4^a^
Na^+^, mEq/L	149 ± 0.9	150 ± 0.9	150 ± 0.9	150 ± 1.2	151 ± 1.2	152 ± 1.9
K^+^, mEq/L	3.8 ± 0.3	3.7 ± 0.3	3.9 ± 0.3	4.4 ± 0.3	4.5 ± 0.3	4.5 ± 0.3
Ca^2+^, mEq/L	3.7 ± 0.1	3.3 ± 0.2	3.3 ± 0.1^a^	3.2 ± 0.1^a^	3.3 ± 0.1^a^	3.4 ± 0.1
Mg^2+^, mEq/L	1.2 ± 0.1	1.5 ± 0.1	1.3 ± 0.1	1.3 ± 0.1	1.3 ± 0.1	1.2 ± 0.1
Cl^-^, mEq/L	130 ± 1.7	130 ± 1.9	134 ± 2.1	136 ± 1.1	139 ± 1.5^a^	145 ± 1.8^a^
Lac^-^, mEq/L	1.5 ± 0.2	5.5 ± 0.9^a^	5.6 ± 0.7^a^	3.7 ± 0.5^a^	2.3 ± 0.4	2.5 ± 0.5
SID_a_, mEq/L	26.1 ± 1.0	24.3 ± 1.5	19.9 ± 2.0^a^	19.0 ± 0.5^a^	19.3 ± 1.6^a^	15.6 ± 1.2^a^
PO_4_^-^, mEq/L	2.5 ± 0.2	3.0 ± 0.2	3.2 ± 0.2	3.4 ± 0.3	3.2 ± 0.4	2.6 ± 0.2
Alb, g/dL	1.5 ± 0.2	1.1 ± 0.1^a^	0.7 ± 0.1^a^	0.7 ± 0.1^a^	0.7 ± 0.1^a^	0.5 ± 0.1^a^
SID_e_, mEq/L	27.8 ± 0.9	19.2 ± 0.7^a^	19.3 ± 0.4^a^	21.6 ± 1.0^a^	21.6 ± 1.0^a^	19.6 ± 0.4^a^
SIG, mEq/L	-2.0 ± 1.5	5.1 ± 2.2^a^	0.6 ± 2.0	-2.6 ± 1.3	-2.2 ± 1.4	-2.9 ± 1.5
AG, mEq/L	3.1 ± 1.2	14.1 ± 3.3^a^	5.8 ± 1.6	2.1 ± 1.2	0.0 ± 1.0	-0.2 ± 1.3
AG_corr_, mEq/L	7.8 ± 1.6	18.6 ± 2.9^a^	8.8 ± 1.8	6.9 ± 1.0	6.6 ± 1.0	5.5 ± 1.2

Data are presented as mean ± standard error of the mean (*n *= 8). ^a^*p *< 0.05 with respect to baseline. AG, anion gap; AG_corr_, corrected anion gap; Alb, serum concentration of albumin; Ca^2+^, plasma concentration of calcium; Cl^-^, plasma concentration of chloride; K^+^, plasma concentration of potassium; Lac^-^, plasma concentration of lactate; Mg^2+^, plasma concentration of magnesium; Na^+^, plasma concentration of sodium; pCO_2_, arterial carbon dioxide partial pressure; PO_4_^-^, serum concentration of phosphate; sBE, standard base excess; SID_a_, apparent strong ion difference; SID_e_, effective strong ion difference; SIG, strong ion gap.

**Figure 3:**
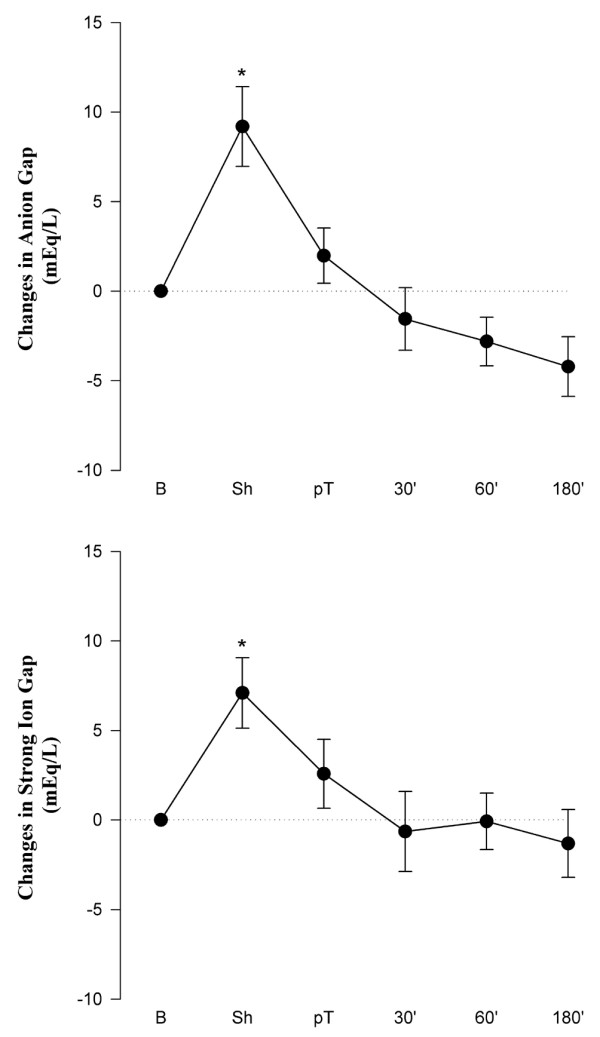
Changes in anion gap (upper panel) and strong ion gap (lower panel) versus baseline at the different measuring points. Absolute values are given in Table 1. Values are mean ± standard error of the mean (*n *= 8). *p < 0.05 with respect to baseline. 30', 60', and 180' indicate time (in minutes) after resuscitation. B, baseline; pT, post-treatment; Sh, shock.

Figure 3 depicts changes in AG and SIG at the different measurement points versus baseline values. After induction of shock, significant increases were observed in AG from 3.1 to 14.1 mEq/L (Δ = +11.0 mEq/L) and in SIG from -2.0 to 5.1 mEq/L (Δ = +7.1 mEq/L). These increases in AG and SIG were only temporary and both returned to near-baseline values after completion of resuscitation. Figure 3 also indicates that a significant correlation existed between AG and SIG (*r*^2 ^= 0.84; *p *< 0.001).

Serum concentrations of all anions determined by means of capillary electrophoresis are given in Table 2. Surprisingly, acetate was found in sera of all dogs at relevant concentrations. Acetate increased from a mean value of 2.4 mEq/L at baseline to a mean value of 4.4 mEq/L after induction of shock and remained elevated until 60 minutes post-therapy. β-Hydroxybutyrate was detected in sera of dogs at concentrations between 1.7 and 2.9 mEq/L but did not change significantly throughout the whole experiment. Sulfate was present in serum at concentrations of approximately 1.4 mEq/L but did not change. Citrate was found in the sera of all dogs, and at baseline, concentrations were approximately 0.5 mEq/L in all animals; serum citrate rose significantly to a mean value of 2.4 mEq/L after induction of shock. Although levels fell from this maximum, they tended to remain elevated during resuscitation. Serum concentrations of fumarate and α-ketoglutarate were below the level of detection at baseline. However, both metabolites were detectable, albeit at low concentrations, after induction of shock and until the end of the experiment. Though present only at negligible concentrations, urate increased significantly versus baseline after shock induction and completion of resuscitation, before gradually returning to normal.

**Table 2:** Analysis of anions by means of capillary electrophoresis

	Time point of measurement					
	Baseline	Shock	Immediately after therapy	30 minutes after therapy	60 minutes after therapy	180 minutes after therapy

Acetate, mEq/L	2.4 ± 0.5	4.4 ± 0.9	5.8 ± 0.4^a^	4.8 ± 0.5	3.9 ± 1.0	2.3 ± 0.5
β-HOB, mEq/L	1.7 ± 0.7	2.0 ± 0.3	1.7 ± 0.2	2.6 ± 1.2	2.9 ± 1.3	2.6 ± 0.9
Sulfate, mEq/L	1.4 ± 0.1	1.5 ± 0.1	1.4 ± 0.1	1.4 ± 0.1	1.3 ± 0.1	1.3 ± 0.1
Citrate, mEq/L	0.5 ± 0.1	2.4 ± 0.7^a^	1.2 ± 0.2	1.3 ± 0.3	1.2 ± 0.2	1.5 ± 0.4
Fumarate, μEq/L	ND	6.2 ± 1.3	6.7 ± 2.2	4.1 ± 1.3	3.7 ± 1.5	5.0 ± 1.3
α-KG, μEq/L	ND	35.3 ± 10.4	25.3 ± 7.9	28.8 ± 4.5	27.8 ± 4.1	20.8 ± 8.5
Urate, μEq/L	15.1 ± 1.1	55.9 ± 14.4^a^	32.7 ± 4.1^a^	26.5 ± 8.5	18.3 ± 2.9	19.7± 6.1

Data are presented as mean ± standard error of the mean (*n *= 4 to 8). ^a^*p *< 0.05 with respect to baseline. α-KG, serum concentration of α-ketoglutarate; β-HOB, serum concentration of β-hydroxybutyrate; acetate, serum concentration of acetate; citrate, serum concentration of citrate; fumarate, serum concentration of fumarate; ND, not detectable; sulfate, serum concentration of sulfate; urate, serum concentration of urate.

**Figure 4:**
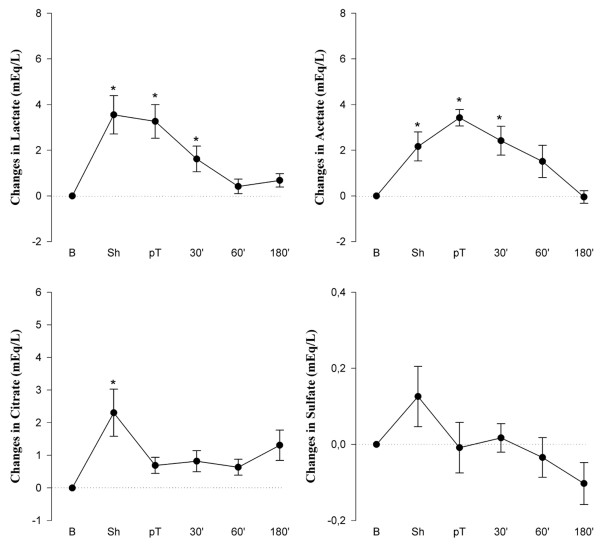
Changes in anions in canine serum at the different measuring points compared with baseline. Absolute values are given in Tables 1 and 2. Values are mean ± standard error of the mean (*n *= 8). *p < 0.05 with respect to baseline. 30', 60', and 180' indicate time (in minutes) after resuscitation. B, baseline; pT, post-treatment; Sh, shock.

Figure 4 shows changes in lactate, acetate, citrate, and sulfate concentrations with respect to baseline values. Notably, the mean increase in serum lactate after induction of shock (Δ = +4.0 mEq/L) accounted for only approximately 36% of the observed increase in AG (Δ = +11.0 mEq/L). After induction of shock, significant and relevant increases in serum concentrations of acetate (Δ = +2.2 mEq/L) and citrate (Δ = +2.2 mEq/L) were found. Despite a slight increase in sulfate after induction of shock, changes in serum concentration of sulfate were small throughout the experiment and, thus, were not responsible for the observed changes in AG and SIG.

**Figure 5:**
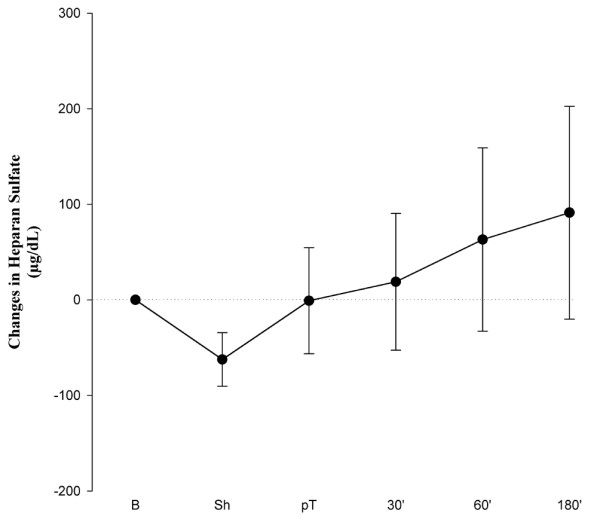
Changes in heparan sulfate concentrations in canine serum versus baseline at the different measuring points. Baseline values were 350 ± 76 μg/dL. Values are mean ± standard error of the mean (*n *= 8). 30', 60', and 180' indicate time (in minutes) after resuscitation. B, baseline; pT, post-treatment; Sh, shock.

Soluble heparan sulfate did not increase during hemorrhage. Levels tended to rise continuously during resuscitation, but the change was not statistically significant (Figure 5). Interestingly, complete hydrolysis of serum with NaOH to liberate organically bound sulfates from glycocalyx constituents such as heparans, chondroitins, and dermatanes failed to markedly elevate the sulfate concentration above the level already present as inorganic sulfate (result not shown).

## Discussion

It has been known for many years that hemorrhagic shock causes metabolic acidosis. In the present model, a prolonged metabolic acidosis associated with a transient increase in AG after shock induction was observed but was not adequately accounted for by the concomitant hyperlactatemia. In addition, the SIG increased significantly after induction of shock.

The physicochemical approach to acid-base balance originally described by Stewart [6] and subsequently modified by Watson [15], Fencl and Rossing [16], and Figge and colleagues [13,17] has become common in the last decade [18-26]. According to this approach, the dissociation equilibrium is supplemented with equations incorporating the necessity for electrical neutrality and the principles of conservation of mass. Weak acid concentrations (albumin and phosphate), the pCO_2_, and the SID have been identified as variables with independent effects on pH [6]. Two different methods of calculating the SID exist. The first, leading to the apparent SID (SID_a_), relies on simply measuring as many strong cations and anions as possible and then summing their charges. The second, yielding the effective SID (SID_e_), estimates the SID from the pCO_2 _and the concentrations of the weak acids [27]. The difference between SID_a _and SID_e _has been termed SIG and attains a positive value when unmeasured anions are present in excess of unmeasured cations and attains a negative value when unmeasured cations exceed unmeasured anions [7].

In the present study, a negative SIG obtained at baseline indicates an excess of unmeasured cations. However, it should be noted that the baseline values were established after surgical preparation and infusion of large amounts of a crystalloid solution, resulting in electrolyte concentrations with particularly high serum chloride levels. Therefore, for graphical depiction, we used relative values representing increments and decrements in SIG and AG.

The data from the present study strongly suggest that large amounts of unmeasured anions, expressed either as the AG or as the SIG, are likely to be generated during states of global tissue hypoxia. This finding is in line with results of Kaplan and Kellum [28], who reported increases in SIG in patients with major vascular injury, a condition generally associated with global tissue hypoperfusion. Also, in a study investigating the cause of the metabolic acidosis after cardiac arrest, Makino and colleagues [29] showed that increases in SIG contributed approximately 33% to the metabolic acidosis.

With regard to the source of unmeasured anions, one can only speculate. An increased SIG appears to occur in patients with renal [30] and hepatic [7] impairment, and unexplained anions have been shown experimentally to arise from the liver in animals challenged with bolus intravenous endotoxin [31]. In our canine model of hemorrhagic shock, serum concentrations of citrate were significantly increased after shock induction. This is in accordance with a recent finding of Forni and colleagues [32], who found elevated levels of anions usually associated with the Krebs cycle in patients with large AG acidosis. Citric acid, a tribasic acid, is reported to be 97% ionized at a pH of 7.0 [33]. Thus, each molecule of citric acid adds three protons to a solution upon ionization, and the contribution of citrate to the generation of unmeasured anions is of much greater significance than is apparent from its molarity.

We believe that the source of citrate is the mitochondria. The rate of oxygen delivery to respiring tissue plays a role in generating citrate, with several authors suggesting that tissue hypoxia can cause an increase in intermediates of the citric acid cycle [33,34]. In further support of the mitochondrial origin of many of the unmeasured anions, fumarate and α-ketoglutarate, both metabolites of the Krebs cycle (like citrate), were identified in the dog sera in this study. Though not detectable by means of capillary electrophoresis at baseline, both metabolites were found in all dog sera after the induction of shock and until the end of the experiment. All of the Krebs cycle intermediates investigated as possible candidates for unmeasured anions have acidic dissociation constants guaranteeing full dissociation at a pH of 7.4 (Table 3).

**Table 3:** pK values of Krebs cycle intermediates

Chemical intermediate	pK_1_	pK_2_	pK_3_
Oxalacetate	2.4	4.4	-
Citrate	3.1	4.8	6.4
Isocitrate	3.3	4.7	6.4
α-Ketoglutarate	3.1	4.4	-
Succinate	4.2	4.4	-
Fumarate	3.0	4.4	-
Malate	3.4	5.1	-

Another potential source of citrate might have been the stepwise reinfusion of whole blood during the standardized induction of shock given that the blood was stored with a CPDA solution. This blood contained approximately 12 mmol citrate per liter, and amounts ranging from 0 to 360 mL were given. However, there was absolutely no correlation between the individually reinfused volume of blood and the level of citrate found afterward in blood samples taken following induction of shock (results not shown). Since citrate changes in serum are a balance between endogenous production, exogenous load, and liver metabolism, a contribution of exogenous citrate to the changes in SIG cannot be ruled out totally.

The rise of acetate during induction of shock is not really surprising. Irrespective of the type of energy-yielding substrate (sugars, amino acids, and fats), oxidative utilization always passes via degradation to acetate, which is then coupled to coenzyme A. Hydrolytic cleavage of acetyl-coenzyme A back to acetate will occur when there is a block in mitochondrial consumption of this thiol-ester. Thus, increased acetate also supports the assumption that mitochondrial dysfunction was caused by the hemorrhagic shock.

Serum concentration of urate also increased significantly after shock induction. This is in excellent agreement with induction of a state of catabolism of high-energy adenine and guanine nucleotides during shock. The rise in urate supports the presumed damage to hepatic metabolism because urate is normally degraded to allantoin in the dog liver. However, the concentrations of this metabolite in dog serum, as befits a non-primate species, were much too low to account for the changes in SIG.

The healthy vascular endothelium is coated by a large variety of extracellular domains of membrane-bound molecules, which together constitute the glycocalyx. Heparan sulfate is a polysulfated polysaccharide that is linked to core molecules of the endothelial glycocalyx. Shedding of these polyanionic heparan sulfates might be another potential source of unmeasured anions, and, indeed, our group has recently demonstrated acute destruction of the endothelial glycocalyx in humans experiencing ischemia and reperfusion injury [35]. The present study also indicates shedding of heparan sulfate after hemorrhagic shock. However, this did not parallel the changes in SIG (Figures 3 and 5). After alkaline hydrolysis of serum, sulfate anions, already present in canine serum at levels of approximately 0.7 mM, did not change enough to account for much of the changes in SIG.

**Figure 6:**
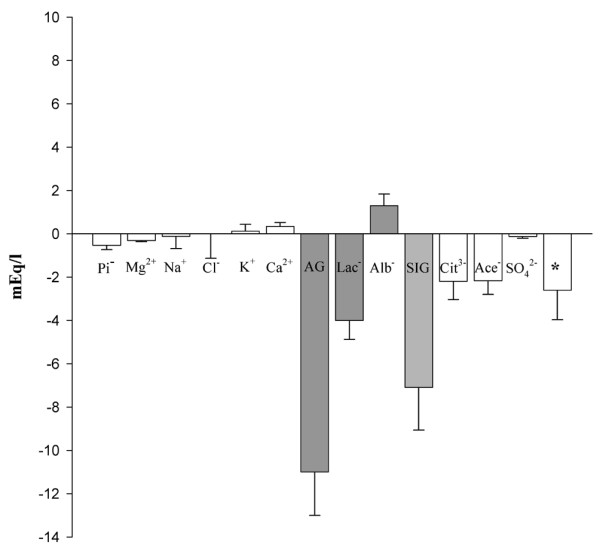
Impact of different variables on the acid-base state during induction of shock. Values (mean ± standard error of the mean) (*n *= 8) are presented as the difference between the time points of baseline and shock. A negative value represents an increase in anionic components or a decrease in cationic components, corresponding to an acidifying effect, and a positive value represents an increase in cationic or a decrease in anionic components, corresponding to an alkalinizing effect. Asterisk indicates unidentified anions that are still missing. Ace^-^, serum concentration of acetate; AG, anion gap; Alb^-^, negative electric charges contributed by albumin; Ca^2+^, serum equivalents of calcium; Cit^3-^, serum equivalents of citrate; Cl^-^, serum concentration of chloride; K^+^, serum concentration of potassium; Lac^-^, serum concentration of lactate; Mg^2+^, serum equivalents of magnesium; Na^+^, serum concentration of sodium; Pi^-^, negative electric charges contributed by inorganic phosphate; SIG, strong ion gap; SO_4_^2-^, serum equivalents of sulfate.

The impact of different variables on the acid-base status during induction of shock is shown in Figure 6. The values represent the difference between the time points at baseline and in shock. Interestingly, changes in AG and SIG were the strongest determinants of acidemia, accounting for -11.0 and -7.1 mEq/L of acidifying effect, respectively. An increase in lactate concentration contributed -4.0 mEq/L to acidemia. Changes in phosphate, magnesium, sodium, and chloride each accounted for less than -0.5 mEq/L of acidifying effect. The acidemia was attenuated by alkalinizing changes of several variables. A decrease in albumin concentration had the strongest alkalinizing effect (+1.3 mEq/L). Increases in potassium and calcium concentration were of minor importance (less than +0.4 mEq/L). The increases in citrate (-2.2 mEq/L), acetate (-2.2 mEq/L), and sulfate (-0.1 mEq/L) concentration together accounted for approximately 63% (-4.5 mEq/L) of the increase in SIG during induction of shock. The net balance yields a deficiency of unidentified anions amounting to approximately 2.6 mEq/L.

Outcome prediction based on the quantitative approach remains controversial. Some investigators have found that the pH and the standard base excess are better outcome predictors than the SIG [36]. However, other investigators have found that the SIG is a powerful predictor of outcome in acutely ill or injured patients. In critically ill patients, SIG was a strong independent predictor of mortality when it was the major source of acidosis [8]. Also, in patients with major vascular injury [28] and in children following cardiopulmonary bypass surgery [27,37], an elevated SIG appeared to be superior to other conventional mortality predictors.

Growing evidence suggests that extracellular acidosis itself has profound effects on the host, particularly in the area of immune function. It is now becoming apparent that different forms of acidosis and even different types of metabolic acidosis produce different effects [38], and SIG generation may be one feature.

Fluid resuscitation might have affected the SIG in the present model of hemorrhagic shock, although only hydroxyethyl starch solutions were given. The colloid molecule itself may be a weak acid. Albumin and gelatin preparations contain a weak acid activity [20,39]. Gelatins have been shown to increase both AG and SIG, most likely due to their negative charge and relatively long circulating half-life [40].

There are several limitations in this study. First, we were not able to find a strict correlation between SIG and the serum concentrations of citrate and/or acetate. This is not entirely unexpected since the generation of the SIG is most probably multifactorial. Second, we have used capillary electrophoresis for identification of potential candidates, and concentrations of still-unknown metabolites may be below the level of detection of this method. Third, using Stewart's approach to acid-base balance has some limitations. A major criticism is a possible inaccuracy of determinations of plasma electrolyte concentrations. Such inaccuracy means that the calculation of the SID_a_, AG, and SIG can be erroneous [41,42]. If, as in our study, mean values of larger collectives are used, always with utilization of the same measurement techniques for determinations of electrolytes, these limitations should be insignificant.

## Conclusion

We demonstrated that large amounts of unmeasured anions were generated after hemorrhage in this highly standardized canine model of shock. Using Stewart's quantitative approach to acid-base balance, we found that the strongest determinant of this acidosis was the SIG. Capillary electrophoresis identified acetate as an important contributor to the SIG. Moreover, we have shown that the serum concentrations of citrate, fumarate, and α-ketoglutarate, all three intermediates of mitochondrial metabolism, were elevated after induction of shock. Sulfate and β-hydroxybutyrate, on the other hand, though present in relevant amounts in serum, did not contribute to the change in SIG associated with hemorrhagic shock. Our study did not assay for a number of other metabolites with potential relevance for acid-base balance as single individual contributors to the unmeasured anions in hemorrhagic shock. These include nitrite, nitrate, oxalate, malonate, oxalacetate, malate, succinate, oxoglutarate, and glucuronate. The amount of sulfate equivalents liberated into plasma as a result of shedding of the endothelial glycocalyx after hemorrhagic shock seems too small to be of quantitative significance. However, taken together, the expected elevations of all these anions in plasma in association with states of hypoxia and shock are undoubtedly significant.

## Key messages

• The present canine model of standardized hemorrhagic shock shows a prolonged metabolic acidosis associated with a transient increase in unmeasured anions after shock induction.

• Capillary electrophoresis suggests that this increase in unmeasured anions is largely attributable to acetate and to anions associated with the Krebs cycle.

## Abbreviations

30' = 30 minutes post-therapy; 60' = 60 minutes post-therapy; 180' = 180 minutes post-therapy; A^- ^= amount of weak plasma acid; AG = anion gap; Alb = serum concentration of albumin; B = baseline; Ca^2+ ^= serum equivalents of calcium; Cl^- ^= serum concentration of chloride; CPDA = citrate, phosphate, dextrose, and adenine; K^+ ^= serum concentration of potassium; Lac^- ^= serum concentration of lactate; Mg^2+ ^= serum equivalents of magnesium; Na^+ ^= serum concentration of sodium; pCO_2 _= partial pressure of carbon dioxide; Phos = serum concentration of phosphate; pT = post-treatment; PVA = polyvinyl alcohol; Sh = shock; SID = strong ion difference; SID_a _= apparent strong ion difference; SID_e _= effective strong ion difference; SIG = strong ion gap.

## Competing interests

At the time these studies were performed, NSF and PEK were full-time employees of Alliance Pharmaceutical Corp. (San Diego, CA, USA), the company that supported the study and provided the perfluorochemical emulsion that was being evaluated in this model of canine hemorrhagic shock. The other authors declare that they have no competing interests.

## Authors' contributions

DB was responsible for acquisition of capillary electrophoresis data, analysis and interpretation of these data, and drafting the manuscript. CJW and KBP helped design, develop, and achieve approval of the original dog shock study, helped perform data acquisition and analysis, took part in all experiments performed, and were responsible for surgical preparation, anesthesia, and data collection. OPH helped design, develop, and achieve approval of the original dog shock study, helped perform data acquisition and analysis, helped plan and design the study protocol, and was involved with ensuring quality data acquisition and analysis through regular audits and site visits. He was the senior investigator who planned, designed, and supervised the whole previous experimental project. FGM helped design, develop, and achieve approval of the original dog shock study and helped perform data acquisition and analysis. PEK and NSF helped design, develop, and achieve approval of the original dog shock study and helped performed data acquisition and analysis. All data interpretation was performed with them, and all manuscripts, including the work presented here, underwent their thorough review and copy-editing. Together with GIK, they planned and designed the study protocol and were involved with ensuring quality data acquisition and analysis through regular audits and site visits. GIK helped design, develop, and achieve approval of the original dog shock study and helped performed data acquisition and analysis. He was responsible for supervision, control of data acquisition, and analysis of the dataset. He was included in data acquisition and analysis as well as in preparation and editing of the manuscript. He was principal investigator and was assisted by FGM at that time. Both prepared, performed, documented, and analyzed every single experiment of the whole series. MJ performed the statistical analysis of capillary electrophoresis. BFB was responsible for study concept and design and for analysis and interpretation of data and helped to draft the manuscript. MR conceived of the study and participated in the data interpretation and manuscript development. DB and GIK contributed equally to this work. All authors have reviewed the manuscript, contributed to its final version, and have read and approved the final manuscript.
